# Qualitative and quantitative analysis of FBN1 mRNA from 16 patients with Marfan Syndrome

**DOI:** 10.1186/s12881-015-0260-4

**Published:** 2015-12-18

**Authors:** Lena Tjeldhorn, Silja Svanstrøm Amundsen, Tuva Barøy, Svend Rand-Hendriksen, Odd Geiran, Eirik Frengen, Benedicte Paus

**Affiliations:** Department of Medical Genetics, Oslo University Hospital, Box 4950, 0424 Oslo, Norway; TRS National Resource Centre for Rare Disorders, Sunnaas Rehabilitation Hospital, 1450 Nesoddtangen, Norway; Department of Cardiothoracic Surgery, Oslo University Hospital, Box 4950, 0424 Oslo, Norway; Institute of Clinical Medicine, Faculty of Medicine, University of Oslo, Oslo, Norway

**Keywords:** FBN1, Marfan syndrome, FBN1 mRNA expression

## Abstract

**Background:**

Pathogenic mutations in *FBN1*, encoding the glycoprotein, fibrillin-1, cause Marfan syndrome (MFS) and related connective tissue disorders. In the present study, qualitative and quantitative effects of 16 mutations, identified in *FBN1* in MFS patients with systematically described phenotypes, were investigated in vitro.

**Methods:**

Qualitative analysis was performed with reverse transcription-PCR (RT-PCR) and gel electrophoresis, and quantitative analysis to determine the *FBN1* mRNA levels in fibroblasts from the 16 patients with MFS was performed with real-time PCR.

**Results:**

Qualitative analysis documented that the mutations c.4817-2delA and c.A4925G led to aberrant *FBN1* mRNA splicing leading to in frame deletion of exon 39 and in exon 39, respectively. No difference in the mean *FBN1* mRNA level was observed between the entire group of cases and controls, nor between the group of patients with missense mutations and controls. The mean expression levels associated with premature termination codon (PTC) and splice site mutations were significantly lower than the levels in patients with missense mutations. A high level of *FBN1* mRNA in the patient with the missense mutation c.G2447T did not segregate with the mutation in three of his first degree relatives. No association was indicated between the *FBN1* transcript level and specific phenotypic manifestations.

**Conclusions:**

Abnormal *FBN1* transcripts were indicated in fibroblasts from patients with the splice site mutation c.4817-2delA and the missense mutation c.A4925G. While the mean *FBN1* mRNA expression level in fibroblasts from patients with splice site and PTC mutations were lower than the mean level in patients with missense mutations and controls, inter-individual variability was high. The observation that high level of *FBN1* mRNA in the patient with the missense mutation c.G2447T did not segregate with the mutation in the family suggests that variable expression of the normal *FBN1* allele may contribute to explain the variability in *FBN1* mRNA level*.*

**Electronic supplementary material:**

The online version of this article (doi:10.1186/s12881-015-0260-4) contains supplementary material, which is available to authorized users.

## Background

Mutations in *FBN1*, encoding fibrillin-1 cause Marfan syndrome (MFS; OMIM #154700) and other heritable connective tissue disorders, referred to as fibrillinopathies [1, 2]. The phenotypes caused by *FBN1* mutations range from isolated, minor manifestations to a lethal, neonatal form of MFS [3, 4]. MFS is an autosomal dominantly inherited disorder, exhibiting variable clinical expressivity [5]. Major clinical manifestations are found in the cardiovascular (aortic aneurysm with dissection), ocular (ectopia lentis), and skeletal systems [6].

*FBN1* (NM 000138.4) contains 65 exons encoding profibrillin-1, a 350 kDa glycoprotein that is processed to fibrillin-1 [7]. Human fibrillin-1 is a main component of 10–12 nm microfibrils located in the extracellular matrix (ECM) of connective tissues [8]. The protein is modular, comprising 47 epidermal growth factor-like (EGF) domains, seven transforming growth factor β (TGF-β) binding protein-like domains, two hybrid domains, and one proline-rich region [9]. Forty-three of the 47 EGF domains are calcium binding domains (cbEGF), of which each is characterised by six cysteine residues, normally forming three disulphide bonds and a calcium binding consensus sequence which is involved in protein structure stabilisation [10].

At the latest update of the UMD-FBN1 mutations database (http://www.umd.be/FBN1/ on 28/08/14), 1847 different mutations and 1096 protein variants have been identified in *FBN1* [www.umd.be/FBN1/] in patients with MFS and a spectrum of related fibrillinopathies [2]. Missense mutations are the most frequent (55 %) type of mutations in *FBN1*, typically affecting cysteins in the highly conserved cbEGF domains [11]. These mutations may be associated with increased proteolytic degradation of fibrillin-1 [12–14]. Twenty-five percent of all known *FBN1* mutations are frameshift or nonsense mutations leading to premature termination codons (PTC) [15, 16], potentially generating truncated fibrillin-1 variants that may assemble into the extracellular microfibrils. Truncated transcripts are usually degraded by the nonsense-mediated mRNA decay (NMD) mechanism [17], which would then result in reduced or no expression of truncated fibrillin-1 thus ameliorating negative effects of microfibrils on ECM. Splice site mutations are also frequent in MFS [18, 19]. Infrequently, large genomic deletions involving single or multiple exons of the *FBN1* gene, as well as whole *FBN1* deletions, have been identified [20–22].

Analyses of fibrillin-1 in cultured dermal fibroblasts from MFS patients have revealed abnormalities in the synthesis, secretion, and deposition of fibrillin-1 in the ECM [23–25]. Further, mutant fibrillin-1 may cause abnormal structure of microfibrils and ECM [26]. Alteration of TGF-β binding protein like domains may play an additional role in the pathogenesis of fibrillinopathies, as increased TGF-β signalling causes deregulation of cytokine function [27, 28]. Two models of MFS pathogenesis have been suggested. According to the haploinsufficiency model, the pathogenesis is based on reduction in the levels of normal fibrillin-1 [22, 29], and according to the dominant-negative model, mutant fibrillin-1 assembles with molecules of the wild-type protein, thereby disrupting the function of ECM [30].

We aimed to investigate qualitative and quantitative effects of 16 mutations in *FBN1* on *FBN1* mRNA in cultured fibroblasts from 16 MFS patients, comparing with fibroblasts from individuals with no known connective tissue disorder.

## Methods

### Patients and cell cultures

In this study we analyzed the *FBN1* mRNA levels in fibroblasts from 16 patients with MFS in whom a presumptive disease-causing mutation has been identified (Table 1). All patients have been systematically examined as previously described [31], and satisfied the diagnostic Ghent criteria from 1996 [32]. The study has been approved by the Norwegian Regional Ethics Committee South-East. Fourteen of the presumptive disease-causing mutations have been reported by us [31]. As controls, fibroblast cultures from four individuals with no known connective tissue disorder were established in Department of Medical Genetics, Oslo University Hospital, and two were commercially obtained (BioNordika, Medprobe, Lysaker, Norway). In order to study intrafamilial variability, *FBN1* expression levels were determined in three first degree relatives of one of the patients.

**Table 1:** *FBN1* genotype, predicted effect, fibroblast *FBN1* expression level, and patient characteristics

*FBN1* nucleotide change	Protein change	Affected domain	*In silico* prediction^a^	*Type of mutation*	*FBN1* mRNA % of controls^b^	Clinical phenotype^c^
c.G629A	p.Cys210Tyr	Hybrid	Probably damaging	Missense	120 ± 35	DOsp
c.G1027A	p.Gly343Arg	TGF-β1	Possibly damaging	Missense	219 ± 35	Dos
c.G2447T	p.Cys816Phe	cbEGF9	Probably damaging	Missense	212 ± 52	DOCsi
c.T2848C	p.Cys950Arg	cbEGF10	Probably damaging	Missense	100 ± 16	DOCsi
c.T4348G	p.Cys1450Gly	cbEGF21	Probably	Missense	169 ± 26	DOoCcSsi
c.T5866C	p.Cys1956Arg	cbEGF29	damaging	Missense	141 ± 24	DOoSi
c.G6388A	p.Glu2130Lys	cbEGF32	Possibly damaging	Missense	119 ± 27	DOSsip
c.G7094A	p.Cys2365Tyr	TGF-β7	Possibly damaging	Missense	109 ± 18	DOS
			Probably damaging			
c.4269_4270delAC	p.Pro1424Argfs*6	cbEGF20	Fs, introducing	PTC (fs)	88 ± 23	DCcSi
c.5559delT	p.Gln1854Lysfs*39	cbEGF27	Fs, introducing	PTC (fs)	75 ± 19	DCcs
c.T6339A^d^	p.Tyr2113X	TGF-β6	PTC skip of exon 25	PTC (fs)	53 ± 8	Ds
c.3083-2A > G	-	Intronic	Skip of exon 25	Splice site	51 ± 10	DOoCcSs
c.4211-1G > A	-	Intronic	Skip of exon 34	Splice site	59 ± 11	DOoCcspi
c.4817-2delA	p.Ile1607_Asp1648del	Intronic	Skip of exon 39	Splice site	80 ± 16	DOCcsi
c.4942 + 2 T > C	-	Intronic	Skip of exon 39	Splice site	72 ± 12	DOoCcSsp
c.A4925G	p.Thr1643_Asp1648del	cbEGF23	CSS	Splice site (CSS)	91 ± 17	DOosi

Exons are numbered according to the reference sequence GenBank NM_000138.4. cb, calcium binding

*EGF*, epidermal growth factor, *fs* frame shift, *PTC* premature termination codon, *TGF* transforming growth factor, *CSS* cryptic splice site

^a^ *In silico* prediction of mutations was obtained using ALAMUT prediction algorithms

^b^Mean *FBN1* mRNA expression levels in five parallel wells of fibroblasts from MFS patients compared to controls (*n* = 6) ± SD. The level in controls was assigned as 100 %

^c^Affection of organ systems is given according to the original Ghent nosology for the diagnosis MFS. Dura mater: Major criterion: D. Ocular system: Major criterion: O, Minor manifestations: o. Cardiovascular system: Major criteria: C, Minor criteria: c. Skeletal system: Major manifestations: S, minor manifestations: s. Skin and integument: Minor criteria implying involvement: i, Pulmonary system: Minor criteria implying involvement: p. ^d^The mutation was contained in the UMD-FBN1 database (recurrent mutation)

Biopsies from forearm skin were grown in complete Chang medium (Sigma-Aldrich, St. Louis, MO) from the same batch, supplemented with L-glutamine, penicillin and streptomycin (Invitrogen, Carlsbad, CA), and incubated at standard conditions (37° C, 5 % CO_2_). Fibroblasts from each individual were cultured in five parallels in a 6-well plate (seeding wells). Cells were harvested at passage number 3–5 in late logarithmic growth phase, assessed by microscopy.

### Reverse Transcription-Polymerase Chain Reaction (RT-PCR)

RNA was extracted from fibroblasts according to the manufacturer’s instruction, using the RNAqueous Small Scale Phenol-Free Total RNA isolation kit (Ambion, Cambridgeshire, UK), and quantified with a NanoDrop ND-1000 Spectrophotometer (NanoDrop Technologies, Wilmington, DE). cDNA synthesis using 600–1200 ng of RNA was performed using the High Capacity cDNA Reverse Transcription Kit (Applied Biosystems, Foster City, CA).

### Qualitative analysis of *FBN1* mRNA

In order to analyse *FBN1* mRNA from patients and control fibroblast cultures, 10 overlapping cDNA amplicons covering the whole *FBN1* mRNA was PCR amplified using cDNA specific primers (Additional file 1). Primers were designed using Primer 3 (www.primer3.sourceforge.net). PCR fragments were resolved by gel electrophoresis using ethidium bromide-containing 2 % NuSieve GTG Agarose gel (Cambrex BioScience, Rockland, ME), which was run at 70 V for 6 h. PCR products were purified using AMPure beads (Beckman Coulter Inc, Brea, CA) and sequenced directly in both directions by ABI PRISM 3730 Genetic Analyzer (Applied Biosystems).

### Quantitative *FBN1* mRNA analysis

*FBN1* mRNA levels were determined by the 7900 HT Fast Real-Time PCR System (Applied Biosystems) using TaqMan Gene Expression Assays for *FBN1* (Hs009731199_m1, Applied Biosystems) and glyceraldehyde-3-phosphate dehydrogenase (*GAPDH*) (Hs99999905_m1, Applied Biosystems). Real-time PCR was performed in triplicates in a 384-well plate and run according to the manufacturer’s recommendation. Negative controls included water and no cDNA template.

Amplification levels of *FBN1* were calculated according to the 2^-ddCT^ method [33] including normalization to the mRNA levels of the house keeping gene *GADPH*, and to the *FBN1* mRNA levels in the six controls.

### Statistical analysis

All results were tested for statistical significance with the two-tailed, unpaired T- test. *P*-values <0.05 were considered statistically significant.

### Computer analysis of mutational effect

Consequences of the investigated mutations were predicted using ALAMUT software (www.interactive-biosoftware.com/alamut/).

## Results

### Qualitative *FBN1* mRNA analysis

**Fig. 1:**
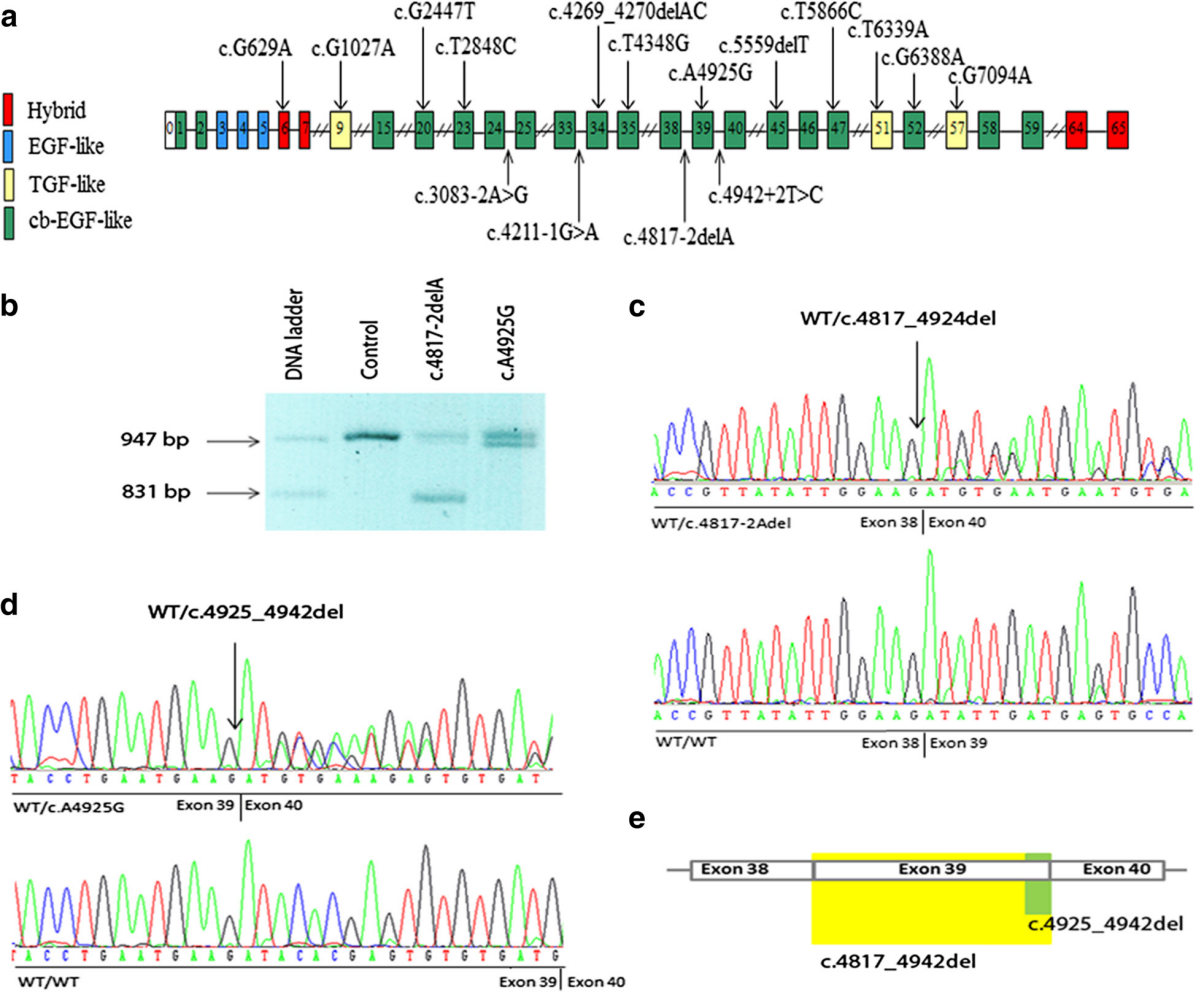
Localization of mutations in FBN1 and results from qualitative analysis (**a**) Schematic presentation of FBN1 with the localization of the 16 mutations investigated in this study indicated (**b**) Analysis of cDNA fragments containing FBN1 exons 36–44 from the patient with c.4817-2delA (lane 3), c.A4925G (lane 4), and control (lane 2). The fragment size marker is in lane 1. Lanes 3 and 4 show fragments corresponding to the normal (955 bp) and truncated cDNA fragments of 829 bp and 937 bp from patients with c. 4817-2delA and c. A4925G, respectively. **c** Fragment of FBN1 cDNA sequence in patient with 4817 + 2 T > C. **d** Fragment of FBN1 cDNA sequence in patient with A4925G. **e** Aberrant splicing resulting from the mutations, c.4817-2delA and c.A4925G lead to two distinct in frame deletions affecting exon 39 (schematic presentation). Skip of exon 39 (c.4817-4942del) (yellow), was confirmed in the patient with the c.4817-2delA splice site variant, whereas deletion of c.4925-4942 (green) was identified in the patient with the c.A4925G variant, which likely introduced a cryptic splice site

Sequence analysis of cDNA from fibroblasts verified the presence of the eight heterozygous missense mutations, c.G629A, c.G1027A, c.G2447T, c.T2848C, c.T4348G, c.T5866C, c.G6388A, and c.G7094A, previously identified in genomic DNA [31]. Ten overlapping amplicons covering the *FBN1* transcript (Table 1) were analyzed by gel electrophoresis and appeared normal in these eight patients (data not shown). Fragment analysis in fibroblasts from patients with premature termination codons (PTCs) (c.4269_4270delAC, c.5559delT, and c.T6339A) did not reveal any aberrant *FBN1* transcripts, and sequencing of the amplicons did not indicate any defects in the mRNA sequence (data not shown). Fragment analysis of *FBN1* mRNA in the patients with splice site mutations (c.3083-2A > G, c.4211-1G > A, c.4817-2delA, and c.4942 + 2 T > C) demonstrated an aberrant transcript in the patient with the c.4817-2delA mutation (Fig. 1a, lane 3) only. Furthermore, the predicted missense mutation, c.A4925G, was shown by fragment analysis to result in an aberrant *FBN1* transcript (Fig. 1a, lane 4). Sanger sequencing of the cDNA amplicon of exons 36–44 from the patients with the c.4817-2delA and c.A4925G mutations showed two in frame deletions, of c.4925-c.4942 (Fig. 1b) and a skip of exon 39 (Fig. 1c), respectively. This suggests that the c.A4925G mutation introduced a cryptic splice site (CSS), resulting in loss of the terminal 18 nucleotides of exon 39 (Fig. 1d).

### Quantitative *FBN1* mRNA analysis

**Fig. 2:**
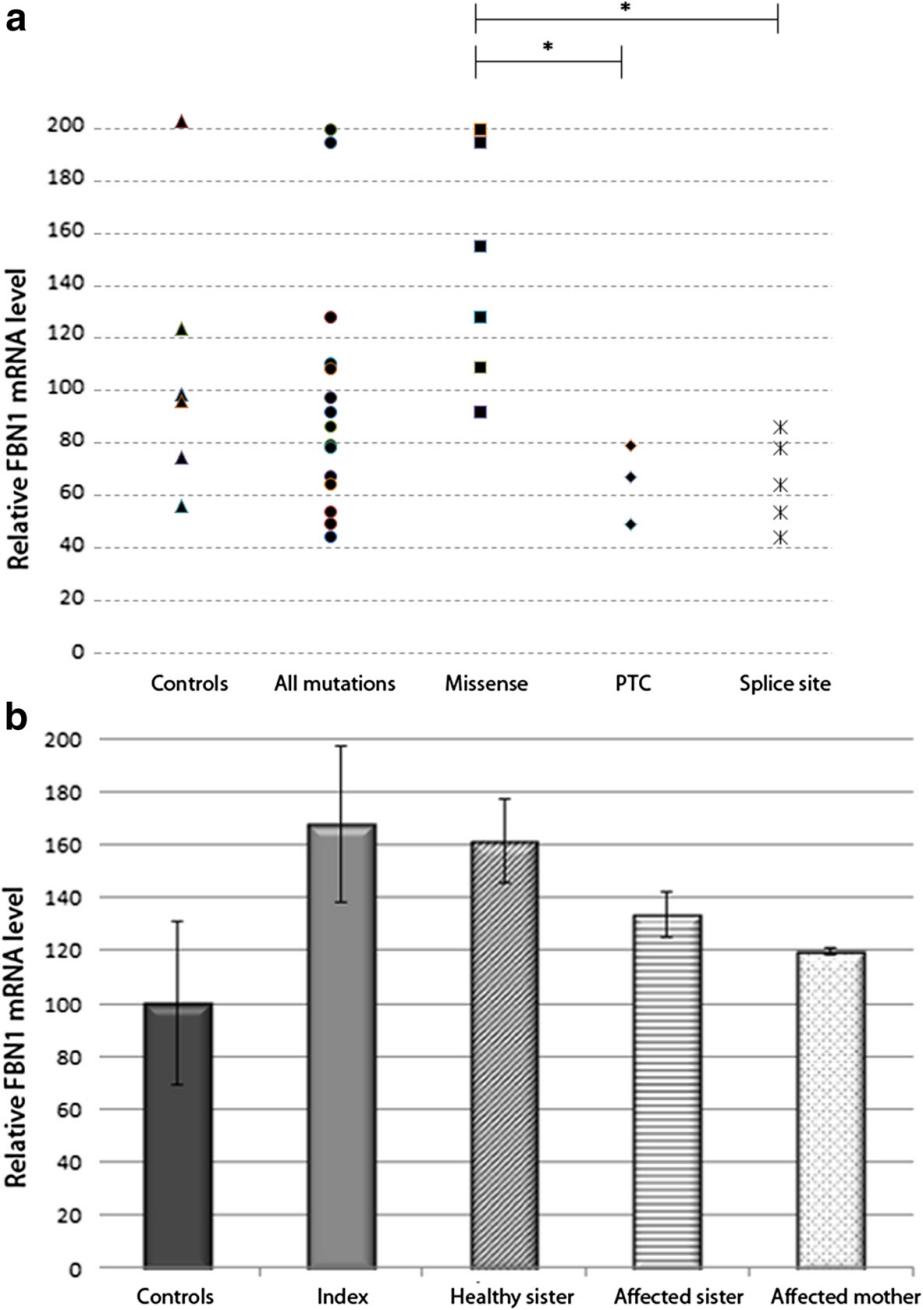
Results from quantitative analysis of *FBN1* mRNA (**a**) *FBN1* transcript levels in fibroblasts from controls (*n* = 6) and MFS patients with missense (*n* = 8), PTC (*n* = 3), and splice site mutations (*n* = 5), respectively. Significant differences (*p* < 0.05) were observed between mean *FBN1* transcript levels in patients with missense mutations and patients with PTC mutations and between the levels in patients with missense mutations and patients with splice site mutations. **b** *FBN1* transcript levels in fibroblasts from the patient with the missense mutation, c.G2447T, his affected mother and sister, both having the c.G2447T, and his unaffected sister, who tested negative for the mutation, compared to the level in controls (*n* = 6). The results are presented as the mean of two experiments with five seeding wells ± SD. The level in controls was assigned as 100 %

Relative *FBN1* mRNA levels in fibroblasts from the 16 patients compared to the level in six controls were determined by qRT-PCR. The mean *FBN1* transcript level in fibroblasts from all 16 MFS patients were not significantly different from controls (Fig. 2). Furthermore, there was no significant difference in the mean *FBN1* transcript level in the group of eight patients with missense mutations, the group of three patients with PTC, or in the group of five patients with splice site mutations compared to controls (Fig. 2a). However, the mean *FBN1* transcript level in the two groups of patients with PTC and splice site mutations were both significantly lower than in the group of patients with missense mutations (Fig. 2a). Individually, the patients with the missense mutations c.G1027A and c.G244T showed significantly higher *FBN1* transcript levels than controls (Additional file 1). To investigate if the high level in the latter patient could be due to the c.G2447T mutation, we investigated if the high *FBN1* mRNA level segregated with the mutation in three first degree relatives; two with the heterozygous c.G2447T and one without. The results showed that all four had higher *FBN1* mRNA levels than the mean level in controls, with the highest level in the index patient and his unaffected sister, who did not have the heterozygous c.G2447T mutation (Fig. 2b).

## Discussion

### Qualitative analysis

Sequencing of cDNA from fibroblasts from the patient with the splice site mutation, c.4942 + 2 T > C did not reveal any abnormal *FBN1* transcript, indicating that the transcript affected by this mutation was efficiently eliminated by NMD. The c.4817-2delA caused a skip of exon 39 in the *FBN1* transcript demonstrated by gel electrophoresis and Sanger sequencing. The fact that the patient had a high *FBN1* mRNA level suggests that most of the *FBN1* transcripts were rescued from NMD. This result is consistent with results from other studies, which have indicated that splice site mutations maintaining the reading frame were not degraded by NMD and therefore did not cause decreased *FBN1* expression [20, 34].

Sequencing of cDNA isolated from the patient with the mutation c.A4925G showed that the mutation predicted to cause the missense p.Asp1642Gly in fact caused the deletion of 18 nucleotides. This finding is explained by introduction of a CSS, which may cause splicing at a position of a transcript where it is usually not spliced [35]. Introduction of CSS has previously been associated with human diseases, including MFS [36, 37].

### Quantitative analysis of *FBN1* mRNA expression

#### *FBN1* mRNA expression in MFS patients and controls

We observed high inter-individual variability in *FBN1* expression levels in fibroblasts from individuals with no connective tissue disorder, as well as in MFS patients (Fig. 2a), similar to results reported by other investigators [38, 39]. Some studies have indicated that *FBN1*expression may be affected by the passage number or different growth conditions for the fibroblasts [26]. In the present study the fibroblasts were cultured under uniform conditions. No trend was observed with respect to expression level compared to age or gender of the skin biopsy donors, the source of the control fibroblasts, or if cells were harvested in different growth phases (data not shown). It has been suggested that the clinical variability in MFS might be explained by varying expression levels of both mutant and normal *FBN1* transcripts [38, 40–42]. In line with this, Hutchinson and co-workers demonstrated that the variable reduction of total *FBN1* transcript in three related individuals carrying a PTC mutation was due to variation in the expression of the normal *FBN1* allele rather than by NMD of mutant RNA [41]. Further, Aubert and co-workers recently carried out differential allelic expression analysis demonstrating reduced *FBN1* transcript levels in patients with PTC and further that 90 % of the transcript originates from the wild type allele [38]. In the present study, the similar high levels of *FBN1* mRNA in both affected and non-affected relatives of the patient with the mutation, c.G2447T indicated that the high level in these family members was not caused by the mutation (Fig. 2b).

### *FBN1* expression, type and location of the mutations

In the present study, eight of the 16 mutations were missense mutations. Our finding of normal or high *FBN1* mRNA levels (ranging from 94 to 219 % of controls) in patients with missense mutations is consistent with findings in other studies [11, 30, 43]. In spite of the high expression level, the MFS phenotype in the patients with missense mutations and high *FBN1* mRNA level could not be considered as mild (Additional file 1). An association between ectopia lentis and missense mutations in the cbEGF domain affecting cysteine residues, in the presence of normal levels of *FBN1* mRNA, has been reported [14]. In the present study, five of the eight missense mutations were located in cbEGF domains of fibrillin-1; four of them affecting cysteine. All four patients with a mutation in cbEGF domain affecting cysteine had ectopia lentis, and their mean *FBN1* mRNA level was 156 % of controls. To our knowledge, few reports exist on the effects of missense mutations in TGF-β binding protein like domains and hybrid domains on *FBN1* mRNA expression. Only one missense mutation in our patients was located in a hybrid domain (also referred to as a TGF-β binding protein like domain); in a moderately affected patient with relatively high *FBN1* mRNA level. Two missense mutations were located in TGF-β1 and TGF-β7 domains, respectively, and the clinical phenotypes of the two patients were rather mild. Their *FBN1* mRNA levels differed two-fold, being 109 to 219 % of controls (Additional file 1). Missense mutations may cause disease through a dominant-negative effect. In line with this, previous studies have demonstrated that missense mutations in *FBN1* caused increased intracellular misfolded fibrillin-1 [11, 30, 43], which is able to cause a severe phenotype in the presence of normal or high *FBN1* mRNA level.

In the two MFS patients with the small deletions c.4269_4270delAC and c.5559delT, the mean *FBN1* mRNA levels were 75 % and 88 % of the level in controls. Both patients had major affection of the cardiac system (Table 1), and both deletions were predicted to cause frameshifts resulting in truncated proteins if the transcripts do not undergo NMD. Analysis by RT-PCR and gel electrophoresis indicated that only the wild type transcripts were present, indicating NMD. These results are consistent with several studies that have shown that nonsense and frameshift mutations in *FBN1* result in efficient NMD [16, 43–45]. However, there are also reports on large out-of-frame deletions of *FBN1* that did not cause reduction of the mRNA levels [21, 22]. Our patient with a nonsense mutation in exon 51 [c.T6339A (p.Tyr2113X)] was clinically moderately affected, although the *FBN1* mRNA level was much lower (53 % of the level in controls) than in the two cases with PTC mutations discussed above (Table 1). Another nonsense mutation in the same codon has been reported to cause exon skipping and NMD of the mutant *FBN1* transcript [15]. This indicates that NMD is activated and is the underlying mechanism causing the low mRNA level in our patient.

The effect of splice site mutations on mRNA is difficult to predict [19, 46]. Four splice site mutations were investigated in this study: the c.4942 + 2 T > C, c.4817-2delA, c.4211-1G > A, and c.3083-2A > G. *In silico* analysis indicated that a skip of exon 39 was likely in the first two cases, and we demonstrated this for the c.4817-2delA. The mutations, c.4942 + 2 T > C and c.4817-2delA were associated with 72 % and 80 % of *FBN1* mRNA expression compared to controls, respectively, and the patients were severely affected with several major, including major cardiovascular, manifestations of MFS (Table 1). The splice site mutations c.3083-2A > G and c.4211-1G > A were associated with 51 % and 59 %, respectively, of *FBN1* mRNA expression compared to controls. These patients were also severely affected (Table 1).

## Conclusions

Qualitative analysis of *FBN1* mRNA from fibroblasts from 16 MFS patients and six controls indicated that the mutations, c.A4925G and c.4817-2delA led to aberrant splicing resulting in frame deletions in exon 39 or deletion of exon 39, respectively. Quantitative mRNA analysis revealed considerable variability in *FBN1* mRNA levels in both MFS patients and controls. No difference in the mean *FBN1* mRNA level was observed between the entire group of cases and controls, nor between the group of patients with missense mutations and controls, but the mean expression levels associated with PTC and splice site mutations were significantly lower than the levels in controls and patients with missense mutations. In line with evidence from other studies, the mRNA levels in fibroblasts derived from four members of one family suggested that variable expression from the normal *FBN1* transcript may contribute to explain the variability in *FBN1* mRNA level.

## Additional file

Additional file 1:
**Primer sequences used for amplification of** ***FBN1*** **cDNA fragments Exons are numbered according to the cDNA sequence in GenBank (accession number: NM_000138.4).** (DOCX 16 kb)
