# Supplementary material for: Qualitative and quantitative analysis of FBN1 mRNA from 16 patients with Marfan Syndrome
Source: BMC Med Genet. 2015 Dec 18;16:113. doi: 10.1186/s12881-015-0260-4 (PMC4683784; doi:10.1186/s12881-015-0260-4)
Supplement: Additional file 1: — Primer sequences used for amplification of FBN1 cDNA fragments Exons are numbered according to the cDNA sequence in GenBank (accession number: NM_000138.4). (DOCX 16 kb) [file 12881_2015_260_MOESM1_ESM.docx]

**Additional file 1: Primer sequences used for amplification of *FBN1* cDNA fragments**

| **Exons** | **Sense** | **Antisense** |
| --- | --- | --- |
| 1-9  9-15  14-23  23-29  28-36  36-44  43-52  50-58  57-64  64-UTR | 5'- GAGGCTGGGAACGTGAAG -3'  5'-CATAGATGTTCGCCCAGGAT -3'  5'-CTGGCATCAGATGGACGTTA -3'  5'-TGAAGTGTTCCCAGGAGTGT-3'  5'-CCCTGGCTACCATTCAACTC -3'  5'- TGCATCAGTGGGAACTGTGT-3'  5'-AGGGGTCTGTGAAAATGGAG-3'  5'-GTGCCTTGAAGGGAGAAGG -3'  5'-GCCGAGGATTCATGACCA -3'  5'-GGGCACTGTGTTTCTGGAAT-3' | 5'-AGTGCTGCTGTGATGCCG -3'  5'-ATGTGAATGCTTCCCTGGAC -3'  5'- TAGGGGGTCATTCAAGTGTCA-3'  5'-CAGAGATCATGCACCGACAT -3'  5'-TATCTGTGACTGCCCACCTG -3'  5'-ATTGACGAGTGTCAGAACGG -3'  5'-AATGCAAAGAACCCGATGTG -3'  5'-TTATTCACGATGTTTGCCGA -3'  5'-AGATCAATGGCTACCCCAAA -3'  5'-TTGTCCATTTATGGTACCTATTTGG -3' |

Exons are numbered according to the cDNA sequence in GenBank (accession number: NM_000138.4)
